# Relaxation dynamics of [Re(CO)_2_(bpy){P(OEt)_3_}_2_](PF_6_) in TEOA solvent measured by time-resolved attenuated total reflection terahertz spectroscopy

**DOI:** 10.1038/s41598-019-48191-4

**Published:** 2019-08-13

**Authors:** Phuong Ngoc Nguyen, Hiroshi Watanabe, Yusuke Tamaki, Osamu Ishitani, Shin-ichi Kimura

**Affiliations:** 1 Department of Physics, Graduate School of Science, Osaka University, Toyonaka, Osaka 560-0043 Japan; 2 Graduate School of Frontier Biosciences, Osaka University, Suita, Osaka 565-0871 Japan; 3 Department of Chemistry, Tokyo Institute of Technology, Meguro-ku, Tokyo 152-8551 Japan

**Keywords:** Infrared spectroscopy, Terahertz optics

## Abstract

To reveal highly efficient photocatalytic properties of an artificial photosynthesis material [Re(CO)_2_(bpy){P(OEt)_3_}_2_](PF_6_), we have directly observed the photo-induced relaxation dynamics and reductive quenching process of the photo-excited state on a photosynthesis material in Triethanolamine (TEOA) solvent as an electron donor by time-resolved attenuated total reflection spectroscopy in the terahertz (THz) region. The spectrum of the complex in TEOA has an intermolecular vibrational mode between the complex and TEOA molecules, which reflects the precursor of the reductive quenching process. The intermolecular vibrational mode has three-step relaxation process in a picosecond timescale after photo-excitation, where firstly the triplet metal-to-ligand charge transfer excited state is vibrationally cooled down, secondly the distance between Re and TEOA is reduced by the rotation of TEOA molecules due to dipole-dipole interaction accelerated by heat transfer, and finally electrons transfer from TEOA to Re. These observations provide us the detailed information of the electron transfer process of photocatalytic properties of [Re(CO)_2_(bpy){P(OEt)_3_}_2_](PF_6_) in TEOA solvent.

## Introduction

Rhenium (I) carbonyl diimine complexes of the type [Re^I^(L)(CO)_3_(N,N)]^n^ (L: monodentate ligand, N,N: diimine ligand) are attracting attention because of the diversity of the photophysical and photochemical behavior of their excited states. They are well-known for their use in photocatalytic CO_2_ reduction^1–5^ and well suited as phosphorescent labels and probes of biomolecules^6–9^. These complexes can be incorporated into supramolecular systems, polymers, or biomolecules, because they are synthetically flexible photo- and redox-active compounds. Moreover, their excited states can be easily tuned by structural variations and the medium^10–12^ such as varying the substituents on either the diimine ligands or the ancillary ligands.

Optically excited states of these complexes have been studied extensively by various spectroscopic techniques^13–15^. Photons with a wavelength of 400 nm excite these complexes from the ground state to the lowest singlet state at the femtosecond timescale. Dynamics of relaxation processes from the singlet excited state that involve fluorescence, intersystem crossing that generates hot triplet states, and phosphorescence on a scale of picoseconds to nanoseconds have been well studied^13,15,16^. Those studies have been mainly performed based on the intramolecular vibrations of CO stretching modes in the IR region, because these modes are sensitive to changes in the electron density on the complexes.

Intramolecular vibrations are used to study the early relaxation dynamics of these complexes and time-resolved fluorescence spectroscopy (Stokes shift) contributes to investigating the relaxation dynamics of solvation^17,18^ and solvent response^19^ to the change in the charge distribution of the solute. Time-resolved IR spectra reflect not only on vibrational cooling^15^ of the triplet metal-to-ligand charge transfer (^3^MLCT) band of the solute and heat transfer into the first solvation sphere^14,17^ but also on the reorganization of the solvent molecules. However, the dynamics of intermolecular interactions between the solute and solvent molecules in the excited state and how the solvent molecules respond to the distortion caused by changes in the solute have not been clarified yet. Moreover, the electron transfer process is one of the most crucial steps in the photocatalytic cycle because it initiates the reduction of CO_2_. The interaction between the catalyst and the electron donor molecules during electron transfer has not been studied so far. Observing the intermolecular interactions between the catalyst and the electron donor molecules when the catalyst in the excited state and during electron transfer will provide understanding of the photocatalytic phenomenon that cannot be explained by investigation of the intramolecular vibrations.

Spectroscopic approaches for studying intermolecular interactions are rather rare because of the very low frequencies of the intermolecular vibrations involved. The intermolecular vibration frequency is typically around the wavenumber of 100 cm^−1^ (~3 THz), sometimes even lower than 50 cm^−1^ (~1.5 THz)^20^ in the THz region. So, THz spectroscopy allows us to access these low-frequency intermolecular vibrations. Most solvents used in photocatalytic studies, however, have a high absorption intensity in the THz region, which suggests that THz transmission spectroscopy is not suitable for conducting such experiments. To overcome this problem, time-resolved attenuated total reflection (TR-ATR) spectroscopy^21,22^ is one of powerful tools for studying molecular interactions upon the excited state in highly absorbing liquids in the THz region as well as for biomolecular studies^23^. Among rhenium (I) carbonyl diimine complexes, [Re(CO)_2_(bpy){P(OEt)_3_}_2_](PF_6_) is one of the most efficient photocatalysts for CO_2_ reduction with high quantum yield for producing CO by excitation with near UV-light^24^.

Here, we report THz spectra of [Re(CO)_2_(bpy){P(OEt)_3_}_2_](PF_6_) in triethanolamine (TEOA) solvent as well as in solid form and its relaxation dynamics in the presence of TEOA as an electron donor by THz time-domain spectroscopy (THz-TDS) and time-resolved attenuated total reflection (TR-ATR) spectroscopy at room temperature. By comparing the ground state spectra for the complex in powder form and in TEOA solvent, we identified the origin of a peak at 1.35 THz as an intermolecular interaction mode of [Re(CO)_2_(bpy){P(OEt)_3_}_2_]^+^ and TEOA molecules. In TR-ATR spectra in TEOA, we observed the three-step relaxation dynamics of the intermolecular vibrational mode at 1.35 THz, where firstly the triplet metal-to-ligand charge transfer excited state is vibrationally cooled down, secondly the distance between Re and TEOA is reduced by the rotation of TEOA molecules due to dipole-dipole interaction accelerated by heat transfer, and finally electrons transfer from TEOA to Re.

## Results

### The ground state

**Figure 1:**
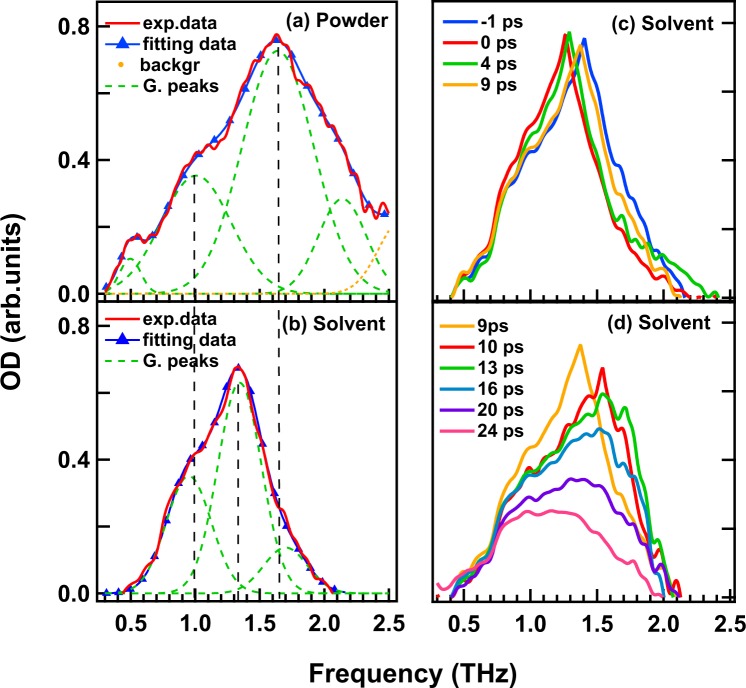
THz spectra of [Re(CO)_2_(bpy){P(OEt)_3_}_2_](PF_6_) (Re complex) powder and that in TEOA solvent, and its time-dependent spectral change. (**a**) THz-TDS spectrum of powder of Re complex. (**b**) THz-ATR spectrum of Re complex in TEOA solvent. (**c**,**d**) Time-dependent TR-ATR spectra of Re complex in TEOA solvent recorded at −1, 0, 4, and 9 ps (**c**) and 9, 10, 13, 16, 20, and 24 ps (**d**) after photo excitation (400 nm, ~70 fs, 6 μJ).

Absorption spectra of [Re(CO)_2_(bpy){P(OEt)_3_}_2_](PF_6_) powder and that in the TEOA solvent obtained by THz-TDS and THz-ATR spectroscopy at room temperature are shown in Fig. 1a,b, respectively. These spectra indicate the ground state (GS) vibrational structure of the powder material and the solvent in the THz region. The broadband absorptions shown in Fig. 1a,b were fitted by Gaussian functions. The spectrum of [Re(CO)_2_(bpy){P(OEt)_3_}_2_](PF_6_) powder displays four broad absorption peaks at 0.5, 1, 1.68, and 2.2 THz and their fitting parameters are shown in Table 1. The peak at 1.68 THz has the highest optical density among all these peaks. Meanwhile, the spectrum of [Re(CO)_2_(bpy){P(OEt)_3_}_2_](PF_6_) in the TEOA solvent has three absorption peaks at 1, 1.35, and 1.7 THz (Table 2 shows these peaks’ fitting parameters) and the peak at 1.35 THz is dominant. Interestingly, when [Re(CO)_2_(bpy){P(OEt)_3_}_2_](PF_6_) powder is dissolved in TEOA, the presence of TEOA molecules causes emergence of the 1.35-THz peak, significant decrease in the intensity of the 1.7-THz peak, and disappearance of the 0.5- and 2.2-THz peaks. However, the 1-THz peak still exists for the solvent, suggesting no effect of TEOA on this absorption.

**Table 1:** Parameters of four absorptions of [Re(CO)_2_(bpy){P(OEt)_3_}_2_](PF_6_) powder obtained by Gaussian fitting.

	Position (THz)	Area	FWHM (THz)
0.5-THz peak	0.500 ± 0.003	0.0130 ± 0.0007	0.130 ± 0.005
1-THz peak	1.000 ± 0.009	0.158 ± 0.006	0.450 ± 0.010
1.6-THz peak	1.640 ± 0.007	0.336 ± 0.014	0.460 ± 0.018
2.2-THz peak	2.140 ± 0.015	0.087 ± 0.007	0.310 ± 0.009

**Table 2:** Parameters of three absorptions of [Re(CO)_2_(bpy){P(OEt)_3_}_2_](PF_6_) in TEOA solvent obtained by Gaussian fitting.

	Position (THz)	Area	FWHM (THz)
1-THz peak	0.950 ± 0.013	0.143 ± 0.007	0.410 ± 0.013
1.35-THz peak	1.340 ± 0.005	0.252 ± 0.015	0.400 ± 0.020
1.7-THz peak	1.700 ± 0.04	0.056 ± 0.009	0.400 ± 0.040

### The excited state

**Figure 2:**
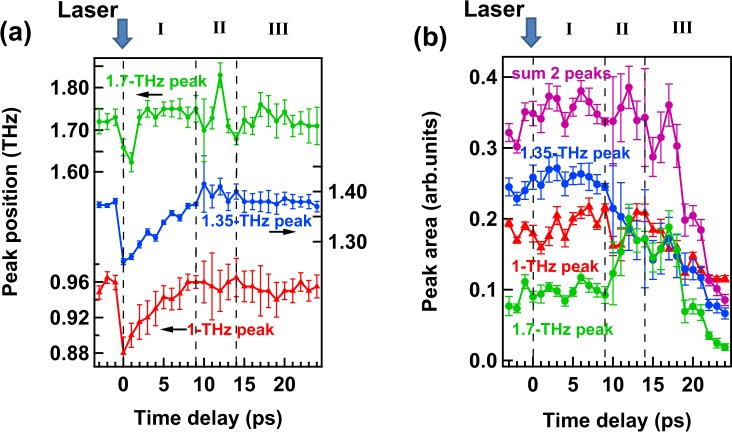
Temporal structure of peak positions (**a**) and peak areas (**b**) of the individual absorption peak at 1, 1.35, and 1.7 THz for [Re(CO)_2_(bpy){P(OEt)_3_}_2_](PF_6_) in TEOA solvent. 1-THz, 1.35-THz, 1.7-THz peaks and the sum of two areas (the 1.35-THz and 1.7-THz peaks) are shown in red, blue, green, and violet, respectively.

TR-ATR experiments were employed to obtain the excited state dynamics in the THz region for [Re(CO)_2_(bpy){P(OEt)_3_}_2_](PF_6_) in TEOA by photo-excitations using 400-nm pulses. The spectral evolutions from −1 to 9 ps and from 9 to 24 ps are shown in Fig. 1c,d, respectively. The spectrum clearly changes after the photo-excitation, where the 1.35-THz peak shifts to the lower-frequency side in 1 ps and returns slowly back to the initial position in 9 ps, as shown in Fig. 1c. From 9 to 24 ps, both spectral shape and intensity change as shown in Fig. 1d. All the spectra are fitted by three Gaussian functions corresponding to 1-THz, 1.35-THz, and 1.7-THz peaks (see Figs S1 and S2 in the Supplementary Information), which are related to the vibrations shown in the absorption spectrum (Fig. 1b). Following that, the temporal development of the peak position and peak area shows a three-step relaxation, as shown in Fig. 2a,b (see Fig. S3 in the Supplementary Information for further information of the temporal structure of the intensity and width of the individual absorption peaks at 1, 1.35, and 1.7 THz). In the first step, the 1-THz and 1.35-THz peaks gradually shift to the higher-frequency side from 0.88 to 0.96 THz and from 1.26 to 1.38 THz, respectively (Fig. 2a). The areas of the two peaks do not change during the time, as shown in Fig. 2b. In the second step, from 10 to 14 ps, the area of the 1.35-THz peak is strongly suppressed and, in contrast, that of the 1.7-THz peak increases. It should be noted that the increase in the peak area of the 1.7-THz peak is as the same amount as the decrease in that of the 1.35-THz peak, which suggests that the sum of these peak area is roughly constant. In the third step, after 14 ps, strong suppression of the 1.35-THz and 1.7-THz peak intensities and slight decrease in the 1-THz peak intensity are observed. The frequencies of the 1-THz and 1.7-THz peaks do not change from 10 to 24 ps.

## Discussion

The origin of the peaks of [Re(CO)_2_(bpy){P(OEt)_3_}_2_](PF_6_) complex in GS can be assigned by the differences between the GS spectra in the TEOA solvent and in powder, shown in Fig. 1a,b. [Re(CO)_2_(bpy){P(OEt)_3_}_2_](PF_6_) complex consists of one cation [Re(CO)_2_(bpy){P(OEt)_3_}_2_]^+^ and one anion PF_6_^−^. In the TEOA solvent, TEOA molecules diffuse into the [Re(CO)_2_(bpy){P(OEt)_3_}_2_](PF_6_) complex and the PF_6_^−^ ions are released from [Re(CO)_2_(bpy){P(OEt)_3_}_2_]^+^ ions, and then the nearest neighbors of [Re(CO)_2_(bpy){P(OEt)_3_}_2_]^+^ ions change from PF_6_^−^ ions to TEOA molecules. Comparing the peaks in the solvent with those of the powder shows that the 1-THz peak still exists with high absorption intensity, suggesting that the origin of the 1-THz peak is an intramolecular vibration mode. Harabuchi *et al*. reported in their vibrational analysis that a vibrational mode^21^ related to the bpy ligand on the [Re(Br)(CO)_3_(bpy)] complex appears at 43 cm^−1^, which is a similar frequency to that observed for the 1-THz peak (1 THz~33 cm^−1^). In contrast, the 0.5- and 2.2-THz peaks disappear and the 1.7-THz peak is strongly suppressed in the TEOA solvent. This can be explained by the replacement of the neighboring PF_6_^−^ ions of [Re(CO)_2_(bpy){P(OEt)_3_}_2_]^+^ ions by TEOA molecules in the solvent, i.e., these peaks originate from the intermolecular interaction of [Re(CO)_2_(bpy){P(OEt)_3_}_2_]^+^ and PF_6_^−^. The emergence of the 1.35-THz peak in the TEOA solvent implies that it originates from the intermolecular vibration of cation [Re(CO)_2_(bpy){P(OEt)_3_}_2_]^+^ and TEOA molecules. These absorption peaks are not observed in TEOA solvent without Re complex (see Fig. S4 in the Supplementary Information).

**Figure 3:**
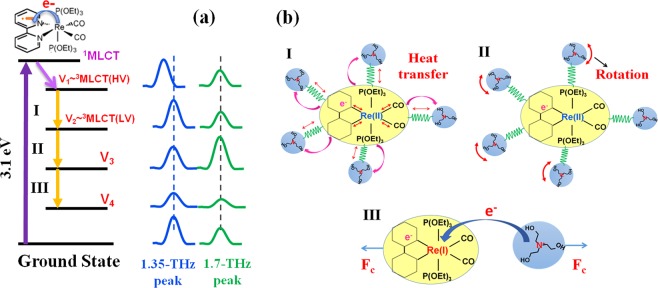
Schematic energy diagram and molecular forms after photo-excitation. (**a**) Schematic diagram of the three-step dynamics from the highest excited state V_1_ to the lowest excited state V_4_ for [Re(CO)_2_(bpy){P(OEt)_3_}_2_](PF_6_) in TEOA solvent in the range of 0.3 to 2.5 THz. (**b**) The schematic figures of the interaction between [Re(CO)_2_(bpy){P(OEt)_3_}_2_]^+^ and TEOA molecules corresponding to dynamical step I-III in (**a**).

The observed temporal structure suggests that electronic and vibrational relaxations with three minor steps exist between the highest excited state V_1_ and the lowest excited state V_4_, as shown in Fig. 3a. The lifetimes from V_1_ to V_3_ are of the order from picoseconds to several tens of picoseconds. Photo-excitation by an optical pulse with the wavelength of 400 nm (3.1 eV) excites electrons from the GS to a singlet metal-to-ligand charge transfer (^1^MLCT) state, in which one electron in the *d* orbital of the Re ion transfers to the π* orbital of the bpy ligand, forming [Re^II^(CO)_2_(bpy^−^•){P(OEt)_3_}_2_]^+^ ^13–17^.

The electron decay to hot triplet excited states (^3^MLCT(HV)) from the ^1^MLCT state simultaneously occurs by ultrafast intersystem crossing (ISC) with a lifetime of a few hundred femtoseconds^13–17^. Since ISC is much faster than the time resolution of this study (>1 ps), the observation made in this study can be assigned to decays occurring after the ^3^MLCT state; thus, the V_1_ state can be assigned to the ^3^MLCT(HV) state. Just after the photo-excitation, all peaks shift to the lower-frequency side and then gradually come back to the initial position in 9 ps. This relaxation timescale agrees with the lifetime of the vibrational cooling from the ^3^MLCT(HV) to the lowest vibrational ^3^MLCT(LV) excited state (4–10 ps) observed by the υ(CO) stretching bands in the IR region^15^. This can be assigned as the first step from V_1_ to V_2_ of the decay process as shown in Fig. 3a, i.e., it is concluded that the relaxation dynamics from V_1_ to V_2_ is the vibrational cooling of the ^3^MLCT band, and the V_2_ level corresponds to be the ^3^MLCT(LV) state. The 1-THz peak shifts in the same manner as the stretching of the intermolecular vibrational mode between [Re(CO)_2_(bpy){P(OEt)_3_}_2_]^+^ and TEOA molecules^15^ as shown in Fig. 3b,I. Therefore, the 1-THz peak can be also assigned to originate from the relaxation of the ^3^MLCT band.

In the V_2_–V_3_ transitional step (second step), from 10 to 14 ps, the time-independent sum of the peak areas of the 1.7-THz and 1.35-THz peaks suggests that the 1.35-THz peak suddenly shifts to the higher frequency of 1.7 THz. This phenomenon can be explained by the reduction of the distance between TEOA molecules and [Re^II^(CO)_2_(bpy^−^•)(P(OEt)_3_)_2_]^+^ ions as shown in Fig. 3b,II. TEOA molecules are supposed to rotate because of the dipole–dipole interaction between TEOA molecules and [Re(CO)_2_(bpy){P(OEt)_3_}_2_]^+^ ions^25^. Indeed, before the photo-excitation, the dipole moments of [Re(CO)_2_(bpy){P(OEt)_3_}_2_]^+^ ions and TEOA molecules are parallel to each other. The dipole direction of [Re(CO)_2_(bpy){P(OEt)_3_}_2_]^+^ ions is changed by the photo-excitation. Hence, just after the photo-excitation, TEOA molecules tend to rotate to reach stabilization against [Re^II^(CO)_2_(bpy^−^•){P(OEt)_3_}_2_]^+^ (equilibrium solvation). The rotation of TEOA molecules is disturbed by other molecules; thus, as shown in Fig. 2, the peak area is constant in region I. In this region, heat transfer occurs from [Re^II^(CO)_2_(bpy^−^•){P(OEt)_3_}_2_]^+^ ions to TEOA molecules and then the temperature of the vibrational modes of TEOA increases. The increase in the temperature of TEOA molecules accelerates the rotation but may not sufficiently. When the vibrational cooling finishes, the temperature of TEOA molecules may be high enough to rotate, then, the sudden jump of the 1.35-THz peak to 1.7 THz occurs. Figure 3b,II displays a schematic figure of the rotation of TEOA molecules in the second step. According to this speculation, we made a model and successfully simulated the reproduction of the experimentally obtained temporal structure. (see Fig. S5 in the Supplementary Information)).

In the V_3_–V_4_ transition (third step), the steep decrease in all the peak areas can be as attributed to the electron transfer process from the nitrogen atom in a TEOA molecule to the Re^II^ center of the Re complex. Indeed, the minimal relative distance between the nitrogen atom of the TEOA molecule and the Re ion causes overlap of their wave function. This effect can allow electron transfer from the TEOA molecule to the Re ion. As a result, the one-electron-reduced species^25,26^ [Re^I^(CO)_2_(bpy^−^•){P(OEt)_3_}_2_] and TEOA^+^• are formed, as shown in Eq. 1 and Fig. 3b,III.

1 $${[R{e}^{II}{(CO)}_{2}(bp{y}^{-}\bullet ){\{P{(OEt)}_{3}\}}_{2}]}^{+}+TEOA\to [R{e}^{I}{(CO)}_{2}(bp{y}^{-}\bullet ){\{P{(OEt)}_{3}\}}_{2}]+TEO{A}^{+}\bullet $$

The repulsive Coulomb force between the Re(I) ion and TEOA^+^• causes a significant reduction in the peak areas of the 1.35-THz and 1.7-THz peaks. Due to the electron transfer, the electronic density of the Re complex changes, which must cause a slight decrease in the intensity as well as the reduction of the 1-THz peak. Referring to the paper by L. M. Kiefer *et al*., the electron transfer from TEOA molecules to the [Re(Br)(CO)_3_(bpy)] complex in an TEOA(20%)/THF(80%) mixed solvent occurs in a few hundred picoseconds^27^. Comparing their experimental conditions with ours, where we used a pure TEOA solvent, suggests that the electron transfer is supposed to occur faster in our case (probably in a few tens of picoseconds). Therefore, the third transitional step in our observation can be reasonably considered to be the electron transfer process.

## Conclusion

To summarize, absorption peaks and their temporal structures of [Re(CO)_2_(bpy){P(OEt)_3_}_2_](PF_6_) in powder form and in TEOA solvent were observed in the THz region by the combination of time-domain spectroscopy and TR-ATR. We assigned the origin of the 1.35-THz peak as the intermolecular vibrational mode between [Re(CO)_2_(bpy){P(OEt)_3_}_2_]^+^ ions and TEOA molecules, the 0.5-, 1.7-, and 2.2-THz peaks as intermolecular vibrational modes of [Re(CO)_2_(bpy){P(OEt)_3_}_2_]^+^ and (PF_6_)^−^ ions, and the 1-THz peak as the intramolecular vibrational mode arising from the bpy ligand as has been reported earlier.

We also revealed three-step relaxation dynamics of the intermolecular vibration between [Re(CO)_2_(bpy){P(OEt)_3_}_2_]^+^ ions and TEOA molecules at 1.35 THz by using time-resolved ATR spectroscopy. Just after photo-excitation, the 1.35-THz peak shifts to the lower-frequency side in 1 ps, and then this peak returns back to the initial position in 9 ps. In the next step, the peak suddenly shifts to 1.7 THz from 10 to 14 ps suggesting a reduction in the distance between the Re ion and TEOA. In the final step, the peak areas decrease after 14 ps, where electron transfer from the nitrogen atom in TEOA to Re occurs and the distance between these molecules is enlarged by the repulsive Coulomb force. The obtained temporal development of the intermolecular vibration at 1.35 THz implies the complex dynamic of the intermolecular interaction between the catalyst and the electron donor solvent. This study provides us detailed information about solute–solvent interactions in photocatalytic activity and helps gain insight into the photocatalysis phenomena.

## Methods

[Re(CO)_2_(bpy){P(OEt)_3_}_2_](PF_6_) powder was synthesized by a method reported previously^28^ using photolysis (>360 nm long-pass filter) of (2,2′-bipyridyl) tricarbonylrhenium trifluoromethanesulfonate in MeCN in the presence of P(OEt)_3_. Reaction with NH_4_PF_6_ in MeOH yielded the title salt, which was subjected to column chromatography on silica with MeCN/CH_2_Cl_2_ (1:5 v/v) as the eluent.

The solute was mixed into TEOA until absorption from the solvent was obtained in the spectrum.

**Figure 4:**
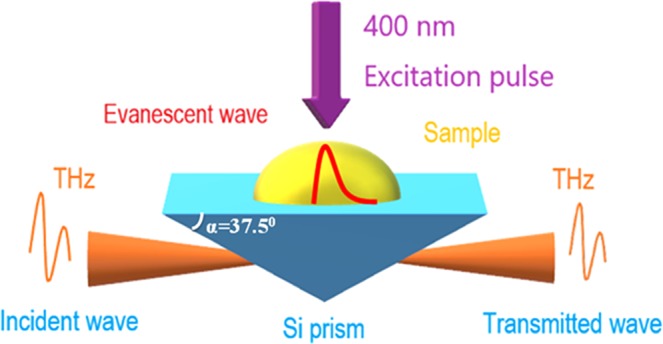
Setup for TR-ATR spectroscopy.

The TR-ATR setup is based on that for conventional time-resolved THz spectroscopy^29^. Importantly, the silicon prism is the core element in the spectrometer as shown in Fig. 4. TR-ATR is obtained by the amplifier of the femtosecond Ti:sapphire laser with an 800-nm center wavelength, 1 kHz repetition, pulse width (FHWM) of ~65 fs and a 900-mW pulse. The laser pulse is split into three pulses, used to generate and probe the THz field and employed as the excitation pulse. One of the three pulses was applied to generate the THz pulse using optical rectification ZnTe (110) 1 mm in thickness. THz pulses were focused by a serial of four parabolic mirrors. The THz spot size of ~3 mm at the sample position was observed by the knife edge method. The Si prism (refractive index n_Si_ = 3.41, base angle α = 37.5°, critical angle θ_i_ = 26°, incident angle θ_1_ = 52.5°) was inserted in the THz path. The THz wave propagating inside the prism approaching the interfaces between the denser medium (Si prism) and the rarer medium (n < n_Si_) induced an evanescent wave. An evanescent wave interacts with the sample, leading to changes in the transmitted THz wave. During the experiment, the sample was placed on the surface of the prism. THz penetrating in free space was detected by electro-optical sampling on ZnTe (110) with a thickness of 1 mm. The spectral range was 0.4 to 2.5 THz with an energy resolution of 0.07 THz. Nitrogen air was purged into the THz beam path to remove absorption of water, although the absorption still appeared in the spectrum at around 1.65 THz. The photoexcitation beam of 6 μJ/pulse, which is the second harmonic of 800 nm, was generated by a BBO crystal (400-μm thickness). The pump beam spot size (~4 mm) was larger than the THz spot size to excite the entire sample. The direction of the photoexcitation beam and TR-ATR setup are illustrated in Fig. 4.

To obtain absorption spectrum of the [Re(CO)_2_(bpy){P(OEt)_3_}_2_]^+^(PF_6_)^−^ powder using THz-TDS^30^, we modified the TR-ATR by removing the Si prism and cutting the pump pulse. The sample was put at the focal point of the THz wave.

## Supplementary information

Supplementary Discussion

## Data Availability

The datasets generated during and/or analyzed during the current study are available from the corresponding author on reasonable request.
