# Supplementary material for: Relaxation dynamics of [Re(CO)2(bpy){P(OEt)3}2](PF6) in TEOA solvent measured by time-resolved attenuated total reflection terahertz spectroscopy
Source: Sci Rep. 2019 Aug 13;9:11772. doi: 10.1038/s41598-019-48191-4 (PMC6692373; doi:10.1038/s41598-019-48191-4)
Supplement: Supplementary file 1 — Supplementary Discussion [file 41598_2019_48191_MOESM1_ESM.docx]

**Relaxation dynamics of [Re(CO)_2_(bpy){P(OEt)_3_}_2_](PF_6_) in TEOA solvent measured by time-resolved attenuated total reflection terahertz spectroscopy**

**Phuong Ngoc Nguyen^A^,** **Hiroshi Watanabe^B,A*^, Yusuke Tamaki^C^, Osamu Ishitani^C^,**

**Shin-ichi Kimura^B,A+^**

^A^Department of Physics, Graduate School of Science, Osaka University, Toyonaka, Osaka 560-0043, Japan

^B^Graduate School of Frontier Biosciences, Osaka University, Suita, Osaka 565-0871, Japan

^C^Department of Chemistry, Tokyo Institute of Technology, Meguro-ku, Tokyo 152-8551, Japan

^*^[hwata@fbs.osaka-u.ac.jp](mailto:hwata@fbs.osaka-u.ac.jp), [^+^kimura@fbs.osaka-u.ac.jp](mailto:+kimura@fbs.osaka-u.ac.jp)

**Supplementary Figures**


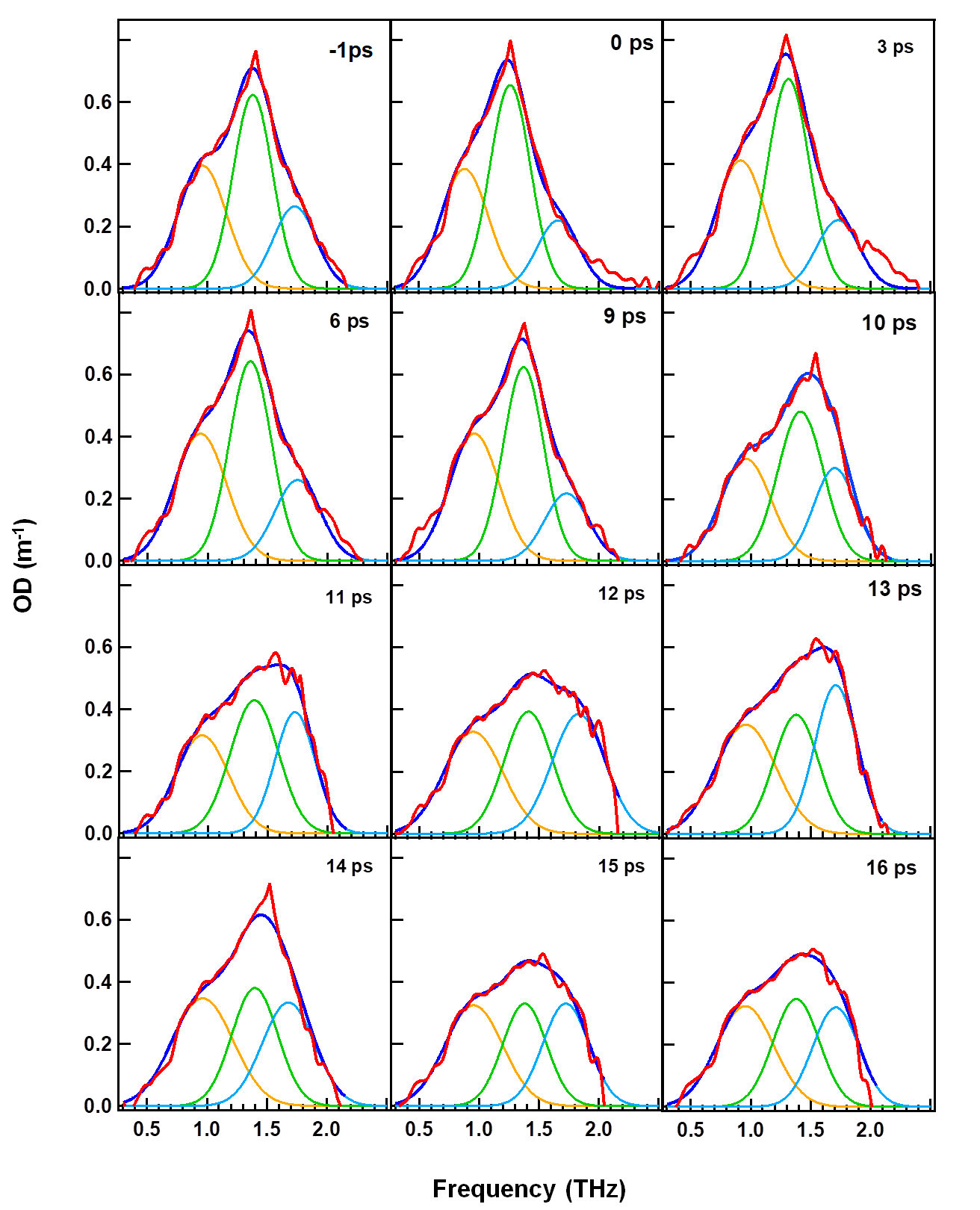


Figure S1: The fitting results of THz-ATR spectra with Gaussian functions $f\left( \omega\right)=\sum_{i=1}^{3} {A_{i}e}^{-\frac{{8ln2\left( \omega-\omega_{oi} \right)}^{2}}{\Gamma_{i}^{2}}}$ at selected time: : -1, 0, 3, 6, 9, 10, 11, 12, 13, 14, 15, 16 ps. Here, $A_{i}$ is the peak intensity,$\omega_{oi}$ the peak position, $\Gamma_{i}$ the peak width (FWHM).The experimental data is shown in red, the sum in blue, the 1-, 1.35, and 1.7-THz peaks in orange, green and skyblue, respectively.


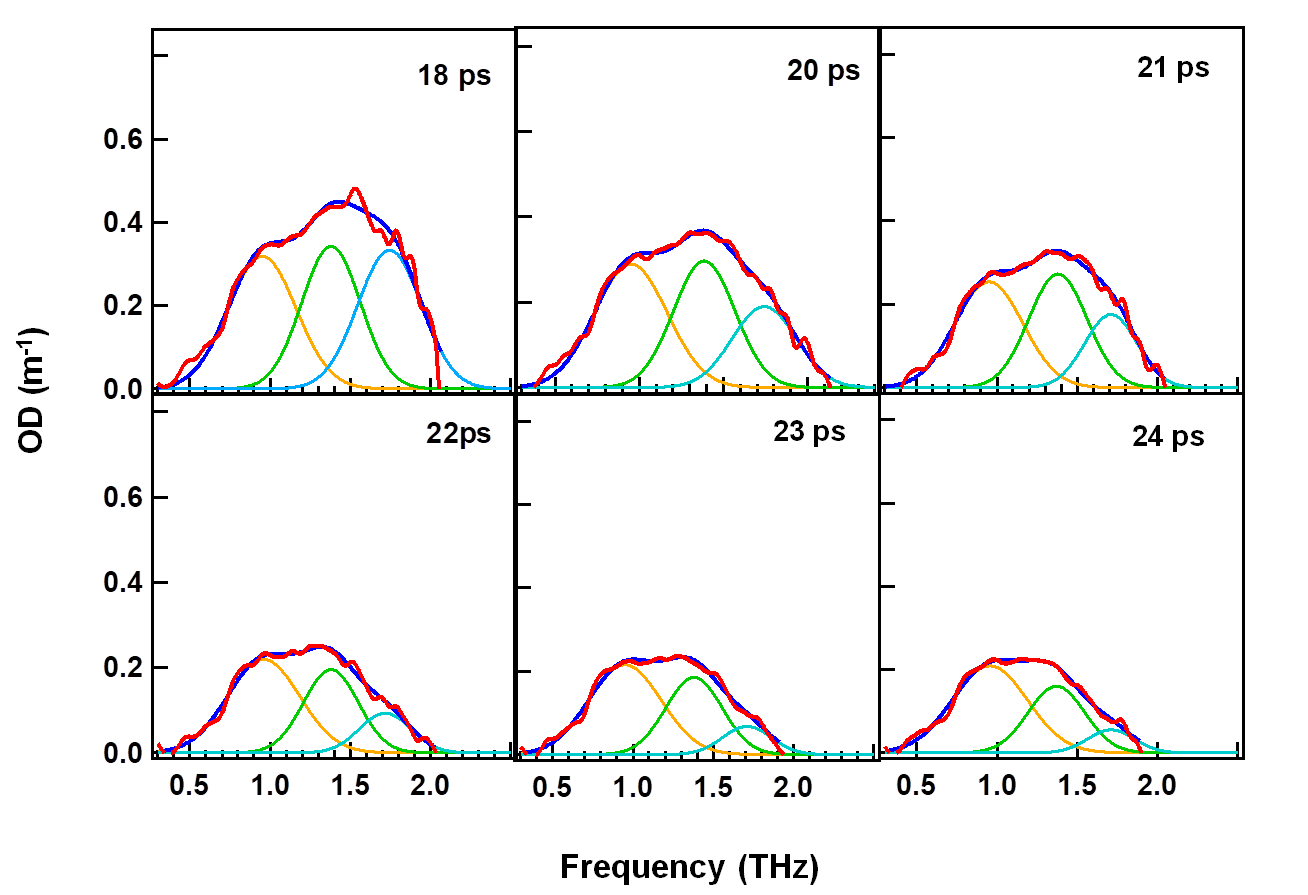


Figure S2: The fitting results of THz-ATR spectra with Gaussian functionsat selected time: 18, 20, 21, 22, 23 and 24 ps. The experimental data is shown in red, the sum in blue, the 1-, 1.35, and 1.7-THz peaks in orange, green and skyblue, respectively.

Figure S3: Temporal structure of peak intensity (a) and peak width (b) of the individual absorption peak at 1, 1.35, and 1.7 THz for [Re(CO)_2_(bpy){P(OEt)_3_}_2_](PF_6_) in TEOA solvent**.** 1-THz, 1.35-THz, 1.7-THz peaks are shown in red, blue, and green, respectively.

Figure S4: Absorption spectrum of TEOA solvent measured by ATR in the range of 0.3 to 2.5 THz.

**Supplementary : Simulation**

As shown in Figure 2 (b), in the V_2_–V_3_ transitional step (second step) in the period from 10 to 14 ps, the peak area of 1.35-THz suddenly shifts to the higher frequency peak at 1.7 THz. This high frequency shift indicates the reduction of the distance between Re complex and TEOA molecule by rotation of TEOA. The rotation should be disturbed by the other molecule, then we propose a model with potential barrier with the barrier energy of *ΔE* as shown in Fig. S5(a). The ΔE decreases by the rearrangement of TEOA which disturbs the rotation. The velocity of the rearrangement is proportional to the square root of the temperature of TEOA (*T_TEOA_*), then the Δ*E* can be described as the eq. S1. The temporal development of both Re complex and TEOA would have the exponentially relaxation as shown in eqs. S2 and S3. The life time of rotation $\tau_{1}$ can be described by using Boltzmann distribution as shown in eq. S4

${\Delta E=E}_{0}-\int_{0}^{t} \alpha\sqrt{T_{TEOA}}dt$ (eq. S1)

$T_{Re}=T_{inc}C_{p}\exp\left( -\frac{t}{\tau_{t}} \right)+RT+T_{inc}$ (eq. S2)

$T_{TEOA}=-T_{inc}\exp\left( -\frac{t}{\tau_{t}} \right)+RT+T_{inc}$ (eq. S3)

$\frac{1}{\tau_{1}}={\beta e}^{-\frac{\Delta E}{k_{B}T_{TEOA}}}$ (eq. S4)

Here, *E*_0_ is the potential barrier energy at *t* = 0 ps, *T_inc_* the increase temperature of TEOA by photoexcitation, *C­_p_* the ratio of the heat capacitance between Re complex and TEOA, *RT* the room temperature (= 300 K), and $\tau_{t}$ the relaxation time of temperature of Re complex and TEOA. Since the effect of the relaxation to the heat bath is considered to be very small in ps time scale, we ignore this effect here. α and β is constant value. Figure S5(b) shows the schematic diagram of the relaxation dynamics after the photo-excitation.

Figures S5(c) and S5(d) show the experimental results and simulation using eqs. S1-S4 with *E*_0_ = 3200 K, *T_inc_* = 100 K, *C_p_* = 2, *α* = 6, *β* = 1000, $\tau_{t}$ = 6.7 ps, and $\tau_{2}$ = 8 ps. After photo-excitation, the heat transfer from a Re complex to TEOA occurs with $\tau_{t}$ = 6.7 ps as shown in Fig. S5 (c). The rotation of TEOA start at 0 ps, but the 1/$\tau_{1}$ is small, because of the low *T_TEOA_* and high Δ*E*. 1/$\tau_{1}$ increases nonlinearly after 9 ps with the increasing *T_TEOA_* and decreasing *ΔE*, as shown in Fig. S5(d). By the rotation, 1.35 THz peak shift to higher frequency of 1.7 THz, and then the electron transfer occurs and finally the 1.7-THz peak intensity decreases in $\tau_{2}$. The model can reproduce the experimentally observed behavior qualitatively. It should be noted that the parameters of *E*_0_, *T_inc_*, *C_p_*, α, β could not be identified from this simulation because many parameters are needed for the fitting. However, this simulation is an example to be able to explain the observed nonlinear behavior.


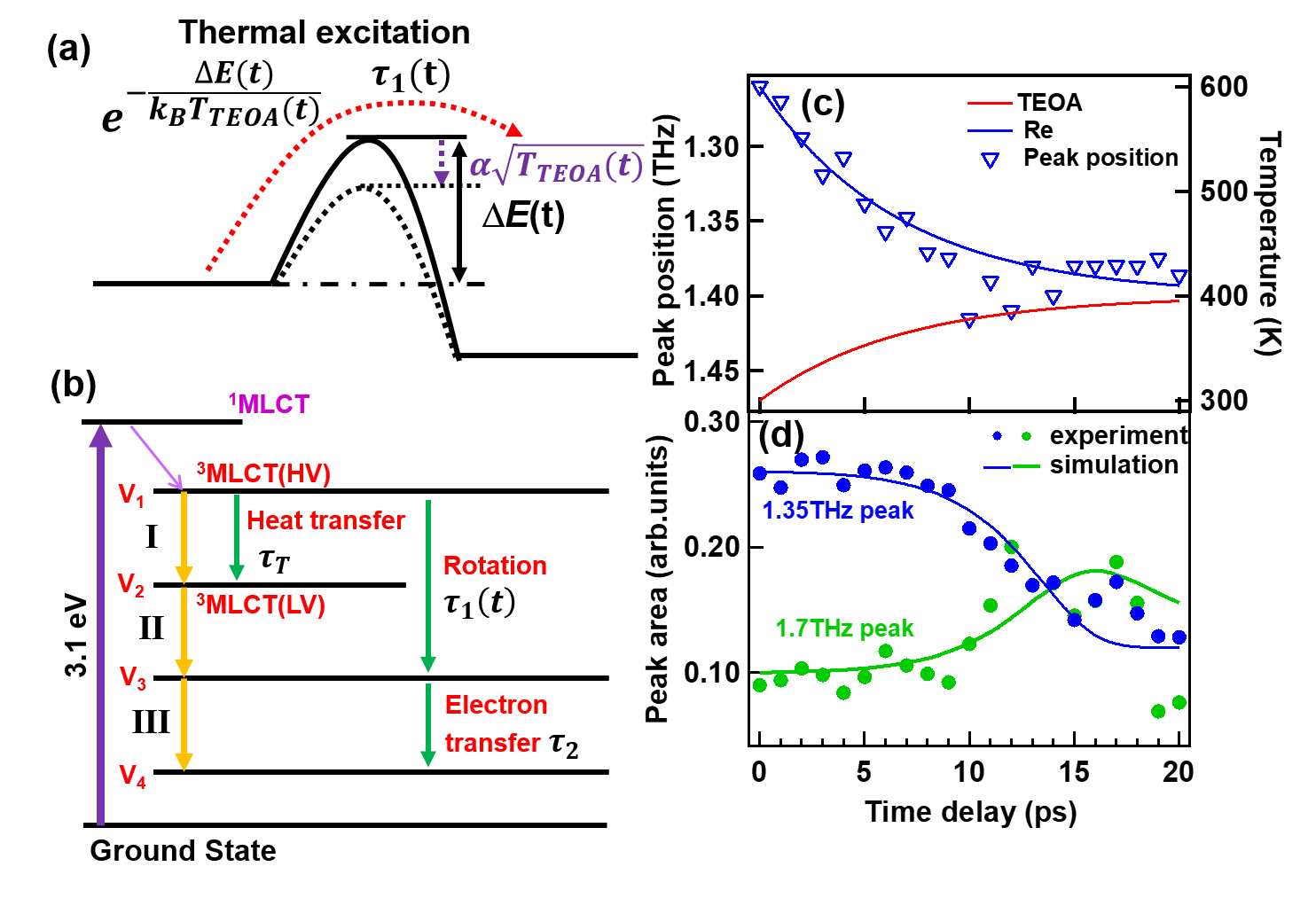


Figures S5 (a) The schematic energy model with a potential barrier of rotation. (b) The schematic diagram of the relaxation dynamics from the highest excited state V1 to the lowest excited state V4 . Comparison of experimental and simulated results. Peak position at 1.35 THz and simulation of Re and TEOA temperature (c) and integrated areas of 1.35-THz and 1.7-THz peaks and simulation of rotation (1.35THz peak decreases and 1.7THz peak increases) and electron transfer (1.7 THz decreases) (d).
